# Antimicrobial Treatment on a Catheter-Related Bloodstream Infection (CRBSI) Case Due to Transition of a Multi-Drug-Resistant *Ralstonia mannitolilytica* from Commensal to Pathogen during Hospitalization

**DOI:** 10.3390/antibiotics11101376

**Published:** 2022-10-08

**Authors:** Junyan Liu, Brian M. Peters, Ling Yang, Hui Yu, Donghua Feng, Dingqiang Chen, Zhenbo Xu

**Affiliations:** 1 College of Light Industry and Food Science, Guangdong Provincial Key Laboratory of Science and Technology of Lingnan Special Food Science and Technology, Innovation Research Institute of Modern Agricultural Engineering, Zhongkai University of Agriculture and Engineering, Guangzhou 510225, China; 2 Key Laboratory of Green Processing and Intelligent Manufacturing of Lingnan Specialty Food, Zhongkai University of Agriculture and Engineering, Guangzhou 510225, China; 3 Department of Clinical Pharmacy and Translational Science, University of Tennessee Health Science Center, Memphis, TN 38163, USA; 4 Department of Laboratory Medicine, The First Affiliated Hospital of Guangzhou Medical University, Guangzhou Medical University, Guangzhou 510120, China; 5 Centre for Translational Medicine, The First Affiliated Hospital of Guangzhou Medical University, Guangzhou Medical University, Guangzhou 510120, China; 6 School of Food Science and Engineering, Guangdong Province Key Laboratory for Green Processing of Natural Products and Product Safety, Engineering Research Center of Starch and Vegetable Protein Processing Ministry of Education, South China University of Technology, Guangzhou 510640, China; 7 Department of Laboratory Medicine, The Second Affiliated Hospital of Shantou University Medical College, Shantou 515041, China

**Keywords:** *Ralstonia mannitolilytica*, chronic obstructive pulmonary disease (COPD), catheter-related bloodstream infection (CRBSI), commensal, pathogen

## Abstract

Despite its commonly overlooked role as a commensal, *Ralstonia mannitolilytica* becomes an emerging global opportunistic human pathogen and a causative agent of various infections and diseases. In respiratory illnesses, including cystic fibrosis and chronic obstructive pulmonary disease (COPD), *R. mannitolilytica* is also identified presumably as colonizer. In this study, one distinctive clone of *R. mannitolilytica* was firstly identified as colonizer for the first 20 days during hospitalization of a patient. It was then identified as a causative agent for catheter-related bloodstream infection with negative identification after effective treatment, verifying its transition from commensal to pathogen. In conclusion, we provide convincing evidence that during hospitalization of a patient, *R. mannitolilytica* transitioned from commensal to pathogen in the respiratory tract leading to catheter-related bloodstream infection (CRBSI).

## 1. Introduction

As a worldwide public health challenge [1], chronic obstructive pulmonary disease (COPD) ranks the third leading cause of death and overall prevalence ranges from 8.6% to 13.6% in China [2,3]. COPD patients present an altered airway microbiome, with *Ralstonia mannitolilytica* identified at a significantly higher rate comparing with a healthy population [4]. Reservoirs of *R. mannitolilytica* in the hospital environment include water [5], saline solutions [6] and oxygen delivery devices [7]. Despite its commonly overlooked role as a commensal, *Ralstonia mannitolilytica* becomes an emerging global opportunistic human pathogen and a causative agent of various infections and diseases, including bacteremia [8,9,10], meningitis [11], sepsis [12], peritonitis [13], osteomyelitis [14], hemoperitoneum [14] and urinary tract infection [7]. Furthermore, *R. mannitolilytica* is also identified in respiratory illnesses such as cystic fibrosis and COPD, presumably as a colonizer [15,16,17,18]. From the first report in 2011, *R. mannitolilytica* isolate G100 was sampled from the sputum of a COPD patient on admission, and the patient was treated with piperacillin/tazobactam [16]. Despite negative identification of *R. mannitolilytica* thereafter, the patient continued to suffer from respiratory failure and eventually died, suggesting this microorganism was a benign colonizer [16,19,20,21]. Such a finding is in accordance with a recent comprehensive study on airway microbiomes, where the presence of *R. mannitolilytica* was occasionally found colonizing COPD patients and indicating its potential role in recurrent infections and diseases [4,22].

For the first time, we report a catheter-related bloodstream infection (CRBSI) case caused by *R. mannitolilytica* in the respiratory tract of a hospitalized COPD patient.

## 2. Results and Discussion

### 2.1. Antimicrobial Agent Treatment

#### 2.1.1. Phase I: From Admission to Day 3

On admission, the patient was diagnosed with acute exacerbations COPD (AECOPD), severe pneumonia and type II respiratory failure, with symptoms of fever and dyspnea on day 1 and high fever and dyspnea on day 2. On day 1, sputum culture identified *Acinetobacter baumannii, Chryseobacterium meningosepticum* and *R. mannitolilytica,* with negative culture from a blood sample. During day 1 to 3, antimicrobial treatment had been conducted using meropenem, vancomycin, caspofungin and voriconazole, with peripherally inserted central venous catheters (PICC) used from day 1 onwards.

#### 2.1.2. Phase II: From Day 4 to 18

Despite antimicrobial treatment during day 1 to 3, the patient failed to recover and a blood test on day 4 revealed an uncontrolled infection accompanied by dyspnea and wheezing. In detail, he had suffered from low fever and wheezing on day 4; low fever, dyspnea and wheezing on day 5; and fever and wheezing on day 6. Consequently, the patient was treated with different antimicrobial therapy (Table 1). Nevertheless, he suffered from intermittent fever. Despite negative blood culture, sputum culture on Day 6 identified *A. baumannii, C. meningosepticum* and *R. mannitolilytica* (Guangzhou-RMAS10/11, and on day 10, only *R. mannitolilytica* was identified), which are common colonizers in the respiratory tract. As antimicrobial treatment was concerned, the patient had been treated with meropenem, vancomycin, caspofungin and amphotericin B during day 4 to 7; meropenem, linezolid, caspofungin and amphotericin B during day 8 and 9; piperacillin/tazobactam, linezolid, caspofungin and amphotericin B during day 10 to 13; cefoperazone/sulbactam, rifampin, vancomycin and amphotericin B during day 14 to 16; and cefoperazone/sulbactam, meropenem, rifampin, vancomycin and caspofungin during day 17 and 18.

#### 2.1.3. Phase III: From Day 19 to 27

On day 19, the patient presented with fever, shivering and dyspnea, and on day 20, his condition deteriorated to high fever, severe dyspnea and septic shock. Remarkably, *R. mannitolilytica* had been identified from both sputum and blood samples (Guangzhou-RMAB10/11/12, three isolates, one from the catheter of PICC and two from peripheral blood) on day 19, validating the occurrence of catheter-related bloodstream infection (CRBSI). According to its antimicrobial profile, ciprofloxacin was used from day 21 on. The patient showed decreased body temperature and improvement in dyspnea, with symptoms of low fever and relieved dyspnea during day 21 to 25, then no fever or dyspnea on day 26 and 27. In addition, a blood test on day 26 revealed normal white blood cell count and serum procalcitonin (PCT), with no positive identification of *R. mannitolilytica* from either blood or sputum samples. Also, PICC was removed on day 27 after the result was reported.

#### 2.1.4. Phase IV: After Day 27 to Discharging

Two months later, this patient presented cough with thick yellow sputum and occasional wheezing. On day 82, the sputum culture identified *Pseudomonas aeruginosa*, *Achromobacter xylosoxidans* and *A. baumannii,* and blood culture identified *Enterococcus faecium* and *A. baumannii*. Consequently, routine treatment for these symptoms was further conducted and the patient was eventually discharged on day 132.

### 2.2. Bacterial Identification

Seven *R. mannitolilytica* isolates were sampled, including four strains (Guangzhou-RMAS10/11/12/13) from sputum and three strains (Guangzhou-RMAB10/11/12) from blood. In detail, *R. mannitolilytica* Guangzhou-RMAS10, Guangzhou-RMAS11, Guangzhou-RMAS12 and Guangzhou-RMAS13 had been recovered from sputum samples on day 1, day 6, day 10 and day 19, respectively. *R. mannitolilytica* Guangzhou-RMAB10, Guangzhou-RMAB11 and Guangzhou-RMAB12 had been obtained from blood samples on day 19. According to the results of 16S rRNA sequencing, compared with all known sequences in GenBank by BLASTn, the sequencing results of all *R. mannitolilytica* strains were found to be identical and clustered at 100% sequence similarity with the *R. mannitolilytica* strain AU255 (AY043379) and AU428 (AY043378) from a cystic fibrosis patient in the United States, and strains LMG 6866 (NR_025385) from cases of nosocomial recurrent meningitis in Belgium.

### 2.3. Antimicrobial Susceptibility Testing

Antimicrobial susceptibility testing (AST) using Vitek2^TM^ Automated System had been performed on seven *R. mannitolilytica* strains [23]. All *R. mannitolilytica* strains shared distinctive antimicrobial resistance profiles, and exhibited resistance to 14 antimicrobial agents, including ampicillin/sulbactam, aztreonam, cefazolin, cefepime, cefoperazone/sulbactam, cefotaxime, cefotetan, cefuroxime, gentamicin, imipenem, meropenem, piperacillin, piperacillin/tazobactam and tobramycin, with only a few susceptible exceptions such as ceftriaxone, ciprofloxacin and levofloxacin. The AST results had been used to determine the antimicrobial treatment on *R. mannitolilytica* strains.

### 2.4. Clonal Relatedness of R. mannitolilytica Strains

**Figure 1:**
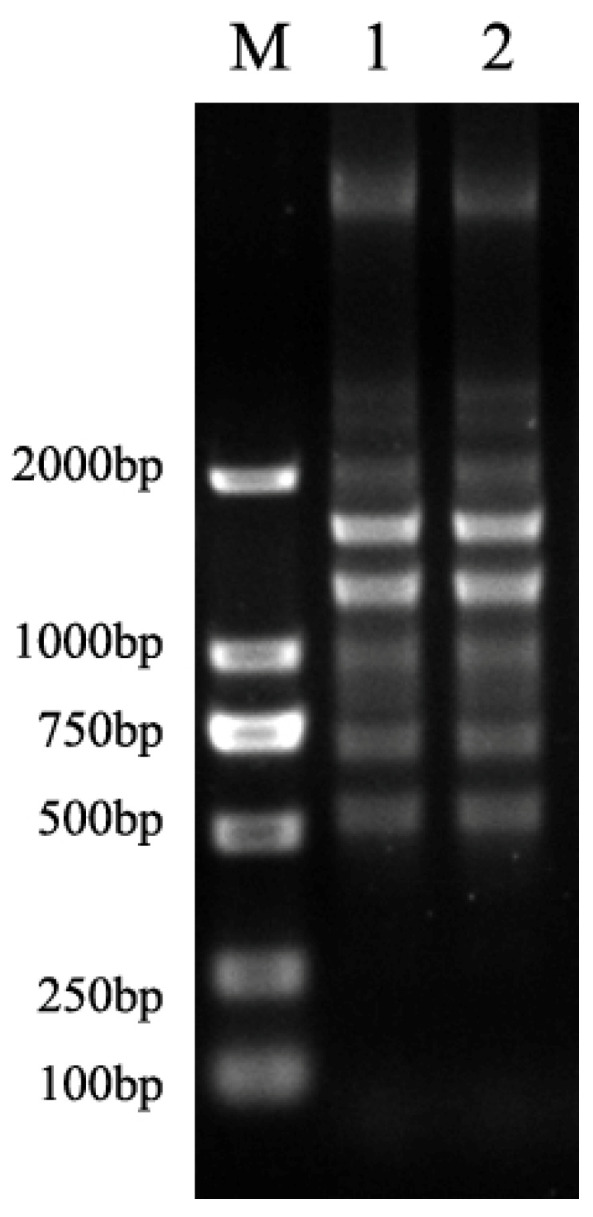
Fingerprinting patterns of two representative strains.

According to the results from randomly amplified polymorphic DNA polymerase chain reaction (RAPD-PCR), the acquired indistinguishable fingerprinting patterns had suggested all seven *R. mannitolilytica* belonged to the same genotype and thus were clonally related (patterns from representative strains shown in Figure 1). As further confirmed by genomic comparison, their identical genomes with similarity higher than 99.99% had convincingly verified that all *R. mannitolilytica* strains originated from a distinct clone (the genome sequences of the distinct *R. mannitolilytica* strain are deposited under accession number CP049132 for chromosome 1 and CP049133 for chromosome 2). In connection with the sampling background, the colonizing *R. mannitolilytica* strain had highly likely induced invasive infection in the respiratory tract and subsequently caused bacteremia in the patient, which explained the identification of clonally related bacteria from sputum and blood culture [24].

### 2.5. Extensive Surveillance on R. mannitolilytica Strains

In addition, from an extensive surveillance during 2014 (including the duration of hospitalization), the *R. mannitolilytica* strain was rarely identified as an infectious agent for other patients, nor was it routinely isolated from drinking water, laboratory-based water, saline solutions used for patient care or catheters [25,26,27].

### 2.6. Transition from Commensal to Pathogen

In this case, rare identification of *R. mannitolilytica* from patients or environmental samples, no recent hospitalization history for the patient, as well as the immediate detection of *R. mannitolilytica* on admission ruled out the likelihood of nosocomial infection or contamination and, thus, highly suggests a community origin of this microorganism. During hospitalization, one identical *R. mannitolilytica* strain was found colonizing the patient’s respiratory tract despite broad antimicrobial therapy (during admission to day 20, shown by four isolates independently sampled from sputum at different time points), and further caused CRBSI (shown by three isolates separately sampled on day 19 including one isolate sampled from the catheter). In addition, the specific treatment of *R. mannitolilytica* (since day 21) correlated with the improvement of symptoms and negative identification of *R. mannitolilytica* (on day 26 and thereafter).

*R. mannitolilytica* has been implicated in acute exacerbation of chronic obstructive pulmonary disease (AECOPD); however, it is still controversial if the bacterium is the causative agent [4,16]. Mostly importantly, this study reported *R. mannitolilytica* bacteremia in a patient diagnosed with AECOPD, confirming its pathogenic role in this disease. Important evidence for this confirmation and verification were as follows: Firstly, for a patient diagnosed with AECOPD, *R. mannitolilytica* strain Guangzhou-RMAS10 was independently isolated from sputum samples (with identical strains) on day 6, but negative for blood samples. Secondly, *R. mannitolilytica* strain Guangzhou-RMAB10 was independently isolated from all three pairs of blood culture bottles on Day 20. Thirdly, *R. mannitolilytica* strains Guangzhou-RMAS10 and Guangzhou-RMAB10 were identical according to the antimicrobial profile, RAPD and genomic sequences. The above evidence strongly indicates the opportunistic pathogen *R. mannitolilytica* induced invasive infection in the respiratory tract (isolates from sputum) and subsequently caused bacteremia (isolates from blood) for this patient. Fourthly, no *R. mannitolilytica* infection for other patients had been reported in the First Affiliated Hospital of Guangzhou Medical University (FAHGMU) in the whole year of 2014 (this bacterium is extremely rare to be reported), and also, during hospitalization of the patient, none of *R. mannitolilytica* were isolated from drinking water, laboratory-based water, saline solutions used for patient care, or catheters. The above evidence and observation highly suggested the unlikelihood of nosocomial infection or contamination as the infection cause. Fifthly, after the confirmation of *R. mannitolilytica* on day 20, the antimicrobial therapy was changed to ciprofloxacin after day 22. Shortly after this, significant improvement from the blood test and negative identification of *R. mannitolilytica* (on day 25 and thereafter) were found. The above evidence presented strong evidence that *R. mannitolilytica* caused bacteremia during the therapy of AECOPD of a patient, as well as its pathogenic role in this disease. In combination with the above evidence, the *R. mannitolilytica* bacteremia in a patient diagnosed with AECOPD, as well as its pathogenicity, is convincing.

## 3. Materials and Methods

### 3.1. Clinical Samples and Bacterial Strains

A total of 16 strains were isolated during the hospitalization of a patient, including 7 *Acinetobacter baumannii* and *R. mannitolilytica* (4 from sputum and 3 from blood samples, sampling on day 1 and 6, and on day 82 (June 22) from both sputum and blood), 2 *Chryseobacterium meningosepticum* (sampling on day 1 and 6), 1 *Pseudomonas aeruginosa* (sampling on day 82 (22 June) from sputum), 1 *Achromobacter xylosoxidans* (sampling on day 82 (22 June) from sputum), and 1 *Enterococcus faecium* (sampling on day 82 (22 June) from blood) strains.

### 3.2. Clinical Case

On 2 April 2014, a 72-year-old male patient presenting with fever and dyspnea was admitted to the First Affiliated Hospital of Guangzhou Medical University (FAHGMU) in Guangzhou, China. With a COPD history dating back to 2004, this patient was treated to relieve symptoms of dyspnea. On admission, the patient was diagnosed with acute exacerbations COPD (AECOPD), severe pneumonia and type II respiratory failure, with symptoms of fever and dyspnea on day 1 and high fever and dyspnea on day 2.

Concerning the clinical history, in January of 2014, the patient was diagnosed with pulmonary aspergillosis and was treated with voriconazole during hospitalization. On 26 February 2014, the patient was hospitalized again due to gastrointestinal bleeding and cured by an antiacid drug. Considering the pulmonary aspergillosis was not cured, the patient was successively treated with voriconazole, mezlocillin sodium/sulbactam sodium, and meropenem in combination with continuous positive airway pressure. On 14 March 2014, the patient was transferred to ICU and successively treated with biapenem, vancomycin hydrochloride, voriconazole and moxifloxacin hydrochloride.

### 3.3. Species Identification

For all samples, bacterial identification was performed with a Vitek2^TM^ Automated System (bioMerieux, Saint-Louis, MO, USA). In addition, all *R. mannitolilytica* isolates were further assessed by sequencing the *16S rRNA* gene [28] using universal primers 27F and 1492R to amplify and then sequence the *16S rRNA* gene, and the obtained sequences were compared to all known sequences in GenBank by BLASTn against the Nr database.

### 3.4. Antimicrobial Susceptibility Testing

Antimicrobial susceptibility testing (AST) was performed with a Vitek2^TM^ Automated System [23]. For *R. mannitolilytica* isolates, 17 antibiotics were used including ampicillin/sulbactam, aztreonam, cefazolin, cefepime, cefoperazone/sulbactam, cefotaxime, cefotetan, ceftriaxone, cefuroxime, ciprofloxacin, gentamicin, imipenem, levofloxacin, meropenem, piperacillin, Piperacillin/Tazobactam and tobramycin (BBI Solutions, Crumlin, UK).

### 3.5. Analysis on Clonal Relatedness of R. mannitolilytica Strains

Random amplification of polymorphic DNA (RAPD) PCR was applied to characterize the genetic relatedness of all *R. mannitolilytica* strains with different origin. The RAPD primer (RM270: 5′–TGC GCG CGG G–3′) was used for random amplification as described previously for *Ralstonia* spp. [29]. Obtaining identical DNA fingerprinting from all 7 *R. mannitolilytica* strains, additionally, genomic sequencing by Illumina HiSeq 2500 (Illumina, San Diego, CA, USA) and genome comparison were further performed for confirmation [30,31].

### 3.6. Extensive Surveillance on R. mannitolilytica Strains

Extensive surveillance on *R. mannitolilytica* was conducted at the same medical setting (FAHGMU) during the whole year of hospitalization (2014).

## 4. Conclusions

In conclusion, we provide convincing evidence that during hospitalization of a patient, *R. mannitolilytica* transitioned from commensal to pathogen in the respiratory tract leading to CRBSI.

## Figures and Tables

**Table 1:** Clinical data of the patient.

			Detection				
	Admission Time	Temperature (°C)	WBC (×10^9^ L)	Neutrophils (%)	Blood Gas: pH/HCO_3_ * (mmol/L) /SpO_2_ * /PaCO_2_ * (mm Hg) /PaO_2_ * (mm Hg)	PCT * (ng/mL)	HCCT *
Phase I	D1	38.3	16.6	77.4	7.42/24.5 /98%/51.2/90.6	0.45	RLLLA *
	D2	38.8–40	20.6	90.1			
	D3	37.5	19.8	94.1			
Phase II	D4		18.3	84.7	7.435/29.1/96.3%/42.8/85.6		
	D5		15.7	82.9			
	D6		15.6	82.3	7.504/26.5/98.8%/33.9/159	4.08	RLLLA
	D7	37.6–38.0	14.6	81.9			
	D8	38.8					
	D9	38.4					
	D10	36.7–38.3					
	D11	38.0					
	D12	38.0	14.3	81.2			
	D13	37.7					
	D14	38.0					
	D15	38.3					
	D16	37.9					
	D17	38.0					
	D18	36.9	16.0	90.7			
Phase III	D19	37.0–38.5			7.346/24.7/98.1%/47.1/152		
	D20	39.4					
	D21	37.9	13.7	75.5			
	D22	37.0–37.9	14.1	88.9			
	D23	38.3					
	D24	37.2–38.3					
	D25	38.0					
	D26	37.1	11.7	89.0	7.365/25.3/98.5%/47.2/119	0.80	Not done
	D27	37.2	9.6	82.7			

* HCCT: High-resolution chest computed tomography. * RLLLA: Right lung lower lobe cavity. * PCT: procalcitonin. * HCO^3^: bicarbonate. * SpO_2_: Saturation of peripheral oxygen. * PaCO_2_: Partial pressure of carbon dioxide. * PaO_2_: Partial pressure of oxygen. Sampling on D1, D6, D10, D19 and D26, and results obtained on D3, D8, D12, D20 and D27, respectively. For blood samples, 3 pairs of aerobic and anaerobic blood culture bottles were collected at different fever spikes, including 1 pair drawn through the catheter and another 2 pairs drawn by bilateral peripheral venipuncture.

## Data Availability

Accession number: CP049132 and CP049133.
